# Diversity affects microclimate temperature and humidity: an overview of the evidence and major unanswered questions

**DOI:** 10.1111/nph.71030

**Published:** 2026-03-05

**Authors:** Alexandra J. Wright, J. English, C. Guimaraes‐Steinicke

**Affiliations:** ^1^ Department of Ecology, Evolution and Behavior University of Minnesota Twin Cities Saint Paul MN 55108 USA; ^2^ Department of Ecology and Evolutionary Biology University of Toronto Toronto ON M5S 3B2 Canada; ^3^ Institute for Earth System Science and Remote Sensing Leipzig University Tal Straße 35 Leipzig 04103 Germany

**Keywords:** biodiversity–ecosystem functioning, climate change, forests, functional traits, grasslands, vapor pressure deficit

## Abstract

Climate change is increasing global temperatures, increasing atmospheric drying, and driving more severe and frequent drought. Plants can cool and humidify microclimates through sensible and latent heat exchange. Higher diversity plant communities can modify microclimates more strongly than lower diversity plant communities, creating the potential for strong biodiversity–climate feedbacks. Here, we review the physical and physiological mechanisms that drive these diversity‐microclimate patterns, catalogue the magnitude of these trends across ecosystem types, and explore how microclimate feedbacks can explain the relationship between biodiversity and ecosystem functioning. We identify key areas where more research is needed (e.g. the role of belowground traits that drive latent heat exchange). This research is essential for understanding how biodiversity and climate are linked at micro‐ and macroscales.

**Table:** 

	Contents	
	Summary	1453
I.	Theoretical underpinning	1453
II.	Evidence for diversity–microclimate temperature relationships	1454
III.	Evidence for diversity–Microclimate RH and VPD relationships	1454
IV.	Major unanswered questions for future work	1456
V.	Conclusion	1457
	Acknowledgements	1457
	References	1458

## I. Theoretical underpinning

Most places on earth are experiencing increasing temperatures, altered precipitation regimes, reduced humidity, and more volatile so‐called ‘hot droughts’ (Yuan *et al*., 2019; IPCC 2023; Novick *et al*., 2024; Gebrechorkos *et al*., 2025). At short timescales and over small spatial scales, vegetation can be used to mitigate the severity of these trends (Wright & Francia, 2024). A substantial body of theory describes the physical and physiological processes through which near‐ground vegetation alters microclimate and governs mass and energy fluxes to the atmosphere (Schulze *et al*., 1994; Geiger *et al*., 1995; McPherson, 2007; Jones *et al*., 2014; Wright & Francia, 2024). Vegetation can (1) shade the understory and reduce irradiative heating, (2) reflect irradiative heating depending on the albedo of the plant surface, (3) increase latent heat exchange due to evapotranspiration, and (4) increase or decrease heat exchange between vegetation and nearby bulk air masses depending on the height and roughness of the vegetation and consequent changes in turbulent air flow. These are linked to particular plant traits and ecosystem types (Wright & Francia, 2024). In particular, the total leaf surface (LAI) determines a canopy's capacity for radiation absorption and evaporative cooling, whereas the vertical three‐dimensional distribution of leaf area density (LAD) controls canopy porosity and the degree of aerodynamic decoupling from the surrounding air (Schulze *et al*., 1994; Jones *et al*., 2014).

Extensions to plant community diversity are much less well studied. Complementary use of niche space aboveground could increase canopy complexity and thus roughness and boundary layer dynamics (Deng *et al*., 2025; Maurer *et al*., 2013). Niche complementarity in root space could also improve water uptake and thus potentially provide more inputs for cooling via latent heat exchange (Guderle *et al*., 2018; O’Keefe *et al*., 2019). These diversity–microclimate effects may also help explain diversity‐driven drought resistance and nutrient cycling (e.g. Tilman & Downing, 1994; Isbell *et al*., 2015; Anderegg *et al*., 2018). To help bridge the gap between mechanistic plant ecophysiology and plant diversity studies, here we review the links between plant diversity and microclimate conditions. We also discuss the connections with climate change and the major open questions in this field.

## II. Evidence for diversity–microclimate temperature relationships

Evidence for a relationship between diversity and microclimate was first collected in experimental North American tallgrass prairie systems. Wright *et al*. (2014) demonstrated that higher diversity grassland communities can be up to 2°C cooler than monoculture systems (with corresponding changes in relative humidity (RH) discussed below). These microclimate effects can feed back and improve performance for small plants growing in high diversity communities on the most arid days (Wright *et al*., 2014, 2015). Since then, an association between plant diversity and microclimate conditions, particularly microclimate temperature, has been demonstrated in many ecosystems globally.

We conducted a formal search of studies that examined the relationship between experimentally established plant diversity and microclimate temperature, humidity, or VPD using Clarivate Web of Science Core Collection and Google Scholar (Supporting Information Fig. S1). This yielded just 23 published studies from 15 diversity experiments that assessed how manipulated plant diversity affected microclimate (Tables 1, S1; Fig. S1).

**Table 1:** We did a formal literature search using Clarivate Web of Science and Google Scholar. This search yielded 23 papers from 15 biodiversity experiments globally.

Paper	Temp	RH	VPD	Sensor type	Sensor location	Diversity Levels	Temp effect	RH effect	VPD effect	Age	Location	Ecosystem type	Experiment name
Schnabel *et al*. (2025)	Yes	No	No	HOBO	Air (1 m)	1 vs 24	+0.4°C at night and autumn, to −2.5°C in July and mid‐day	−	−	10 yr	China	Forest	BEFChina
Martin‐Guay *et al*. (2022)	Yes	No	No	Thermocouple	Soil	1 vs 4	0	−	−	10 yr	Quebec, Canada	Forest	IDENT McGill
Gottschall *et al*. (2019)	Yes	No	No	HOBO	Soil	1 vs 5	0	−	−	12 yr	Germany	Forest	Kreinitz Tree Diversity Experiment
Heinecke *et al*. (2024)	Yes	No	No	Lascar Easylog	Air & Soil	1 vs 4	0 to −1°C (depending on composition)	−	−	10 yr	Belgium	Forest	FORBIO
Aguirre *et al*. (2021)	Yes	Yes	Yes	iButton	Air (12 cm)	0 vs 8	0	4%	0	2 yr	CA, USA	Grassland	Bio3D
Whittington *et al*. (2013)	Yes	No	No	iButton	Soil	1 vs 32	0 to −1°C (depending on season)	−	−	15yr	MN, USA	Grassland	BigBio
Wragg (2015)	Yes	Yes	Yes	iButton	Air (10 cm) & Soil (1 cm)	1 vs 16	−0.5°C in air to −2°C soil	−	−0.15kPa	19 yr	MN, USA	Grassland	BigBio
Guimarães‐Steinicke *et al*. (2021)	Yes	No	No	TLS	Leaf	1 vs 8	0	−	−	4 yr	Germany	Grassland	Jena TBE
Wolf *et al*. (2017)	Yes	No	No	Infrared gun	Soil	2 vs 16	−4°C	−	−	4 yr	CA, USA	Grassland	Zavaleta
Huang *et al*. (2024)	Yes	No	No	PT100 Resistor	Soil (5–15 cm)	1 vs 60	+1.5°C at night to −4.5°C in the summer at mid‐day	−	−	18 yr	Germany	Grassland	Jena Main
Cowles *et al*. (2016)	Yes	Yes	Yes	iButton	Air (10–25 cm)	1 vs 16	−1.5°C	−	−1.8 kPa	20 yr	MN, USA	Grassland	BigBio
Wright *et al*. (2014)	Yes	Yes	Yes	iButton	Air (20 cm)	1 vs 16	−2°C	13%	−0.7 kPa	14 yr	MN, USA	Grassland	BioCON
Lundholm *et al*. (2010)	Yes	No	No	Soil temp probe	Soil (1 cm)	1 vs 15	−2.5°C	−	−	2 yr	Halifax, CA	Herbaceous Green roof	Lundholm
Ramirez & Wright (2023)	Yes	Yes	Yes	iButton	Air (5–10 cm)	1 vs 3	0	0	0	4 months	CA, USA	Crop System	Ramirez

Of these, only 14 papers reported mean microclimate effects associated with experimentally manipulated species richness (full literature results in Supporting Information Table S1). Here, we show these 14 papers as well information about what sensor was used in the study (Sensor Type), where microclimate was measured (Sensor Location), whether temperature, relative humidity (RH), or vapor pressure deficit (VPD) were measured, the levels of plant diversity that were set by the experimenters (Diversity Levels), and the mean level of microclimate increases (+) or decreases (−) in temperature, RH, and VPD. We also provide information about how many years the experiment was running when microclimate was measured (age), the location of the experiment, the type of ecosystem where it took place, and the name of the experiment.

We can use this to make very cursory conclusions about diversity and microclimate temperature relationships for grasslands and forests. Specifically, higher diversity temperate grasslands in Europe, Minnesota (USA), and California (USA) can cool soil temperatures by over 4°C during the warmest conditions, and warm soil temperatures by 1°C at night (Wolf *et al*., 2017; Huang *et al*., 2024) and higher diversity tree communities can cool soil temperatures by up to 2.5°C in the middle of the day and during summer, and can warm microclimates by 0.5°C at night (Zhang *et al*., 2022, 2024; Schnabel *et al*., 2025). These studies also show large variation in how microclimate is measured (soil, air, understory sensor surface, and canopy leaf surface), which will result in very different predictions about leaf warming vs understory warming, and air mixing (e.g. Schulze *et al*., 1994 vs Meinzer, 1993). Despite this, there are some consistent diversity effects reported across measurement types and ecosystem types (Table 1).

More recent work has attempted to unravel the functional traits and community properties that drive diversity–temperature relationships (Wright & Francia, 2024). Guimarães‐Steinicke *et al*. (2021) demonstrated that the negative effects of grassland diversity on microclimate temperatures are most strongly associated with greater canopy complexity and greater allocation of plant biomass across vertical strata (likely affecting roughness length and boundary layer mixing). Some portion of diversity effects on microclimate temperature is likely related to greater biomass in higher diversity communities (Tilman *et al*., 2001; Cowles *et al*., 2016). Plant biomass and plant functional diversity also have independent and co‐occurring impacts on microclimate conditions (Böhnert *et al*., 2016; Wright *et al*., 2021; Wright & Francia, 2024). Greater leaf area and canopy cover in higher diversity ecosystems may contribute to shading effects as well as leaf transpiration effects, and thus also mediate some portion of the diversity–temperature relationship (Zhang *et al*., 2022; Schnabel *et al*., 2025). Finally, traits that lower microclimate temperatures due to evaporative cooling are also potentially operating more efficiently in higher diversity ecosystems, although this includes a great number of species traits and community properties (e.g. evaporation rates, stomatal conductance, and sap flow) that are largely unexamined at this point (but see Caldeira *et al*., 2001; Bruner *et al*., 2023).

In nonexperimental systems, naturally occurring higher diversity forests can indirectly contribute to microclimatic cooling through higher stand structural complexity (Ehbrecht *et al*., 2017), which in turn may offset high daytime maximum temperatures (Terschanski *et al*., 2024). Tree diversity within palm plantations can cool understory air temperatures by 2.5°C (Donfack *et al*., 2021). Outside of forests, naturally occurring species‐rich grasslands can reduce microclimate temperature maxima compared with lower diversity areas (Wang *et al*., 2025) and similar patterns have been observed for lichen‐shrub associations in tundra ecosystems (Mallen‐Cooper *et al*., 2021). Herrera (2025) showed that individual dryland species (thistles) produced unique microclimate temperature signatures in response to daily temperature fluctuations. While these authors did not look at diversity *per se*, examining functional traits that influence thermoregulatory processes may help us scale up to diversity–microclimate patterns.

## III. Evidence for diversity–microclimate RH and VPD relationships

A positive relationship between plant diversity and humidity has been suggested for nonvascular plant communities since at least 2001, but has not been examined as extensively as microclimate temperature relationships. Mulder *et al*. (2001) demonstrated that individual mosses and liverworts survived better when growing in higher diversity communities during drought. The authors suggested (but did not measure) humidity to explain these patterns (see also Caldeira *et al*., 2001 in grasslands).

**Fig. 1:**
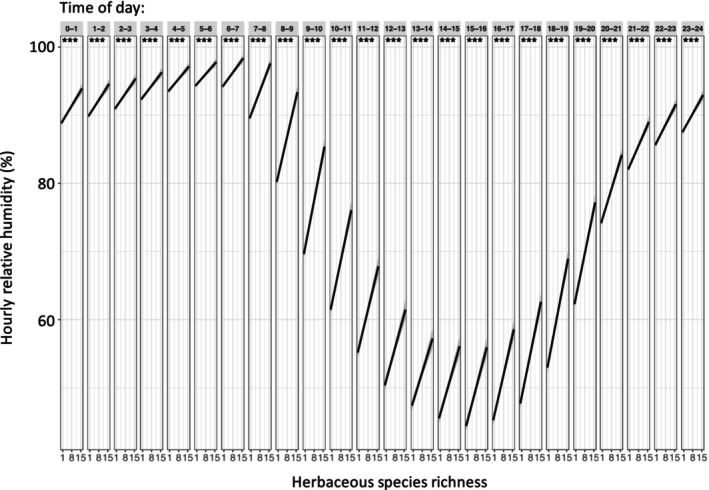
We measured relative humidity every 5 min during the growing season in the Biodiversity, CO_2_, and Nitrogen (BioCON) experiment in central Minnesota in 2012 (Reich *et al*., 2001, Wright *et al*., 2015). Sensors were mounted 10 cm above the soil surface within the plant canopy. We averaged all humidity measurements within each hour of the day and used ordinary least squares regression to test how plot‐level species richness influenced hourly microclimatic humidity (panels represent hour of day). We found that herbaceous species diversity affects humidity of the microclimate at every point during a 24‐h cycle, although this is stronger in the middle of the day where the slope of the line is steeper.

In temperate grasslands, higher diversity communities can, on average, have a RH which is 10% higher than monocultures, and VPD can be over 1.5 kPa lower on the hottest and driest days (Wright *et al*., 2014, 2015; Table 1; Fig. 1). This is equivalent to the difference in VPD between the temperate deciduous forests of Minnesota, USA, and the arid shrublands of New Mexico, USA (Ficklin & Novick, 2017). In our search of the literature, there were no equivalent values published for higher diversity tree communities, although greater canopy cover (associated with diversity) can reduce daily VPD variability by 0.75 kPa in a temperate tree diversity experiment in Minnesota, USA (Zhang *et al*., 2022; Park *et al*., 2025). There are only five studies that have reported on any aspect of RH or VPD in any biodiversity experiment world‐wide, despite indications of strong diversity‐dependent humidity patterns (Table 1; Fig. 1).

Examinations of RH in observational diversity gradients are incredibly rare. There is evidence of an association between primary productivity, normalized difference vegetation index (NDVI) (which may be associated with diversity), and reduced microclimate VPD (Bernath‐Plaisted *et al*., 2023), but no explicit examinations of species richness, trait composition, or trait diversity on microclimate VPD or RH in natural systems. Wright *et al*. (2021) demonstrated that higher diversity experimental grasslands have an average of 5% higher RH than lower diversity grasslands, even when biomass in higher diversity plots is *lower* than in monocultures. This is also not driven by a sampling effect for individual ‘humidifier’ species: the individual species with the greatest effects on humidity were not even included in the most humid high diversity communities. There may thus be strong emergent effects of diversity on microclimate humidity and VPD (Wright *et al*., 2021).

There has also been a large focus on diversity and microclimate humidity following the conversion of high diversity tropical rainforests to monoculture or low diversity oil palm plantations (Böhnert *et al*., 2016; Donfack *et al*., 2021). Under these circumstances, high diversity tropical forests in Indonesia can have up to 30% higher RH than converted monoculture oil palm and rubber plantations (Böhnert *et al*., 2016). However, these effects are confounded by large biomass changes, and thus, it is impossible to extract biomass effects from diversity effects in these observational systems.

## IV. Major unanswered questions for future work

The future of plant diversity–microclimate research is wide open. We know there are diversity‐dependent patterns affecting temperature, RH, and VPD, but we mostly do not know how they work, the scale of the effects, or how large the implications may be for constituent species growing in these systems (e.g. microclimate–ecosystem functioning). Considering this, we break our suggestions into two categories:

### Mechanisms underlying diversity–microclimate relationships

The next generation of microclimate research should focus on the main mechanisms or traits that underlie the positive effect of diversity on microclimate temperature and humidity. We know that biodiversity can lead to increased productivity, and both diversity and productivity can control microclimate temperature and humidity (Wright *et al*., 2021; Wright & Francia, 2024). But within this, there are major unanswered questions such as:

1. *Are microclimate temperature and humidity in higher diversity plant communities more strongly controlled by direct heat exchange or indirect (latent) heat exchange?* For example, for higher diversity communities with greater canopy complexity or leaf area index, is shading the most important driver of reduced temperature and increased humidity? For some ecosystem types (and growing conditions), it might not be. Grasslands during drought can sometimes cool leaf surface temperatures more than forested systems, likely due to a riskier stomatal strategy when soil water is limiting (van Heerwaarden & Teuling, 2014). Forests can shift to conservative water‐use strategies more quickly than grasslands, reducing water loss and prolonging soil moisture availability (van Dijke *et al*., 2023). This could mean that during drought, grasslands are more strongly controlled by latent heat exchange than by sensible heat exchange. Cooling under shrubs may also be tightly linked to transpiration rates (Bruner *et al*., 2023).
2. *Are higher diversity ecosystems better at latent heat exchange than lower diversity ecosystems?* Do higher diversity communities have complementary water‐use strategies that allow them to evapotranspire more water and drive down microclimate temperatures? Higher diversity plant communities can have greater rooting depth complementarity that can lead to increased resource consumption in the rooting layers (Caldeira *et al*., 2001; Tilman *et al*., 2001, but see Bachmann *et al*., 2015). Seasonal complementarity can also control soil water acquisition, as early‐growing species can sometime preempt soil water for later‐season species (Nord & Lynch, 2009), and sometimes compartmentalize soil water to allow for coexistence. Greater consumption and utilization of belowground water resources could lead to greater rates of evapotranspiration and consequent effects on microclimate temperature and humidity.
3. *How does the boundary layer affect canopy leaf surface temperatures vs understory air vs understory leaf surface temperatures, and how does this interact with plant diversity?* Thicker or rougher boundary layers can affect air exchange between the microclimate and the nearby bulk air mass, and thus modified microclimates could be better ‘trapped’ in ways that benefit (or are detrimental to) constituent species (e.g. Meinzer, 1993). How does roughness length in higher diversity communities affect leaf surface temperatures vs understory temperatures across ecosystem types?
4. *Albedo: for ecosystems that tend to have higher albedo leaves (e.g. gray leaves in arid shrublands), do diversity effects depend more on albedo than surface area?* In such systems, is it more important to have higher albedo leaves than to have higher leaf area coverage? Is there complementarity in leaf albedo and other reflective vs absorptive leaf traits that drives stronger cooling effects in higher diversity communities (e.g. Wang *et al*., 2025)?
5. *Traits and remote sensing: can microclimate conditions and traits associated with microclimate conditions be cheaply monitored or measured at large spatial scales?* Monitoring and assessing microclimate using remotely sensed products can be particularly challenging due to the high spatial and temporal variability of conditions above, within, and below a plant canopy (Körner & Hiltbrunner, 2018; DeFrenne *et al*., 2025). At local scales, statistical ecological studies predicting above‐canopy microclimate have demonstrated that basic principles of turbulent air mixing over canopy surfaces can effectively estimate latent and sensible heat exchange. However, conditions below the canopy remain poorly understood (Maclean *et al*., 2021 but see Bernath‐Plaisted *et al*., 2023). More work is needed here to reconcile the roles of species traits, diversity, and the mismatch between coarsely measured variables (from satellites) and fine‐resolution variability in microclimate.

### Implications of diversity–microclimate relationships

A final point for consideration is whether cooler temperatures and higher relative humidity in higher diversity plant communities result in greater productivity, nutrient cycling, or other indicators of improved ecosystem functioning (Gottschall *et al*., 2019; Beugnon *et al*., 2024; Zhang *et al*., 2024). There is evidence that greater plant diversity can lead to altered nutrient cycling due to changes in understory temperature and VPD (Joly *et al*., 2017; DeGroote *et al*., 2018; Gottschall *et al*., 2019; Zhang *et al*., 2024). In particular, diversity‐driven changes in temperature explained 30% of decomposition rates in a tropical tree diversity experiment in China (Seidelmann *et al*., 2016). In a temperate deciduous forest experiment, diversity‐driven VPD explained 4.3% of leaf litter mass loss rates across species (Zhang *et al*., 2024).

There is also evidence for microclimate mediating diversity–stability relationships during drought (Isbell *et al*., 2015; Anderegg *et al*., 2018; Liu *et al*., 2022). The vast majority of diversity–stability work has focused on how complementary resource use and selection for drought‐tolerant species (that compensate for losses experienced by drought‐sensitive species) can explain stability patterns. However, given that we have evidence for higher diversity grasslands and forests being cooler and more humid than lower diversity communities, and the magnitude of this effect is stronger during periods of drought (Wright *et al*., 2014), could microclimate modulation help explain diversity‐drought patterns? Individual species growing in higher diversity systems can be protected from severe drought stress (Wright *et al*., 2015; Aguirre *et al*., 2021), and this can scale to improved overall growth for some sensitive species or life stages (Wright *et al*., 2014; Aguirre *et al*., 2021). We do not know how widespread these patterns are, or whether they scale to whole community stability (but see Park *et al*., 2025). Examining this question in the context of both soil moisture reductions and dry air manipulations is essential (Wright & Collins, 2023), as the microclimate effect is tied more strongly to air moisture than soil moisture (and both are common during naturally occurring droughts).

## V. Conclusion

While biodiversity and microclimate studies have started to unravel these patterns, much more research is needed. For the future of biodiversity research, we recommend that researchers measure microclimate variables (particularly RH and VPD) across biogeographic systems and canopy positions, focus on teasing out underlying functional mechanisms using experiments, and further explore how microclimates could help explain the much more well examined relationship between biodiversity and ecosystem functioning.

## Competing interests

None declared.

## Author contributions

AJW wrote the first draft of the manuscript in conversation with JE and CGS. JE conducted the database search. All authors contributed equally to revisions.

## Disclaimer

The New Phytologist Foundation remains neutral with regard to jurisdictional claims in maps and in any institutional affiliations.

## Supporting information


**Fig. S1** PRISM diagram of literature search that was conducted for Table 1.
**Table S1** Full list of manuscripts identified by literature search as well as reference information for those not mentioned in the main text.
Please note: Wiley is not responsible for the content or functionality of any Supporting Information supplied by the authors. Any queries (other than missing material) should be directed to the *New Phytologist* Central Office.
