# Supplementary material for: Diversity affects microclimate temperature and humidity: an overview of the evidence and major unanswered questions
Source: New Phytol. 2026 Mar 5;250(3):1453–9. doi: 10.1111/nph.71030 (PMC13062697; doi:10.1111/nph.71030)
Supplement: Supplementary file 1 — Fig. S1 PRISM diagram of literature search that was conducted for Table 1. Table S1 Full list of manuscripts identified by literature search as well as reference information for those not mentioned in the main text. Please note: Wiley is not responsible for the content or functionality of any Supporting Information supplied by the authors. Any queries (other than missing material) should be directed to the New Phytologist Central Office. [file NPH-250-1453-s001.docx]

**Supplementary Materials**

**New Phytologist Supporting Information**

**Article title:** Diversity affects microclimate temperature & humidity: an overview of the evidence and major unanswered questions

**Authors:** Wright, Alexandra J.^1^, English, J.^2^, Guimaraes-Steinicke, C.^3^

**Article acceptance date: 28 January 2026**

***Figure S1.*** *We conducted a formal search of the published literature in Web of Science Core Collection and also Google Scholar ("plant diversity" OR "herbaceous diversity" OR "tree diversity" OR "shrub diversity" (All Fields)) AND (microclimate AND (temperature OR “relative humidity” OR VPD) (All Fields)) AND ("diversity gradient" OR "planted diversity" OR "diversity experiment*" OR "complementarity effect" OR "diversity manipulation" OR "biodiversity experiment*" (All Fields)). This yielded 28 non-duplicate records which we combined with 7 publications we knew about a priori but were missed by the screening (two are preprints and the others don’t use common language to describe the experimental diversity component). After screening, this yielded 35 published studies. Of these 12 were removed because they didn’t experimentally manipulate diversity, or the causal relationship was in the opposite direction (microclimate affects diversity), or non-plant diversity was manipulated. This left us with 23 studies that have examined an experimental or causal relationship between diversity and temp, humidity, or VPD, and only 14 that published averages as a function of the diversity manipulation.*

| ***Authors*** | ***Temp*** | ***RH*** | ***VPD*** | ***Sensor Type*** | ***Sensor Location*** | ***Diversity Levels*** | ***Temp Effect*** | ***RH***  ***effect*** | ***VPD***  ***effect*** | ***Age*** | ***Location*** | ***Ecosystem Type*** | ***Experiment Name*** |
| --- | --- | --- | --- | --- | --- | --- | --- | --- | --- | --- | --- | --- | --- |
| ***Schnabel et al. 2025*** | ***yes*** | ***no*** | ***no*** | ***HOBO*** | ***Air (1m)*** | ***1 vs 24*** | ***+0.4ºC at night and autumn, to -2.5ºC in July and mid-day*** | ***-*** | ***-*** | ***10 years*** | ***China*** | ***Forest*** | ***BEFChina*** |
| ***Martin-Guay et al. 2022*** | ***yes*** | ***no*** | ***no*** | ***Thermocouple*** | ***Soil*** | ***1 vs 4*** | ***0*** | ***-*** | ***-*** | ***10 years*** | ***Quebec, Canada*** | ***Forest*** | ***IDENT McGill*** |
| ***Zhang et al. 2022*** | ***yes*** | ***yes*** | ***yes*** | ***Lascar Easylog*** | ***Air & Soil*** | ***1 vs 4*** | ***No mean values*** | ***No mean values*** | ***No mean values*** | ***10 years*** | ***Belgium*** | ***Forest*** | ***FORBIO*** |
| ***Gottschall et al. 2019*** | ***yes*** | ***no*** | ***no*** | ***HOBO*** | ***Soil*** | ***1 vs 5*** | ***0*** | ***-*** | ***-*** | ***12 years*** | ***Germany*** | ***Forest*** | ***Kreinitz Tree Diversity Experiment*** |
| ***Wang et al. 2025*** | ***yes*** | ***no*** | ***no*** | ***Lascar Easylog & TLS*** | ***Air & Leaf*** | ***1 vs 4*** | ***No mean values*** | ***-*** | ***-*** | ***10 years*** | ***Belgium*** | ***Forest*** | ***FORBIO*** |
| ***Heinecke et al. 2024*** | ***yes*** | ***no*** | ***no*** | ***Lascar Easylog*** | ***Air & Soil*** | ***1 vs 4*** | ***0 to -1ºC (depending on composition)*** | ***-*** | ***-*** | ***10 years*** | ***Belgium*** | ***Forest*** | ***FORBIO*** |
| ***Seidelmann et al. 2016*** | ***yes*** | ***yes*** | ***no*** | ***HOBO*** | ***Air*** | ***1 vs 24*** | ***No mean values*** | ***No mean values*** | ***No mean values*** | ***5 years*** | ***China*** | ***Forest*** | ***BEFChina*** |
| ***Wu et al. 2021*** | ***yes*** | ***yes*** | ***no*** | ***HOBO*** | ***Air*** | ***1 vs 16*** | ***No mean values*** | ***No mean values*** | ***No mean values*** | ***6 years*** | ***China*** | ***Forest*** | ***BEFChina*** |
| ***Wang et al. 2025*** | ***yes*** | ***no*** | ***no*** | ***HOBO*** | ***Air (1m)*** | ***1 vs 24*** | ***No mean values*** | ***No mean values*** | ***No mean values*** | ***10 years*** | ***China*** | ***Forest*** | ***BEFChina*** |
| ***Coyne et al. 2025*** | ***yes*** | ***no*** | ***no*** | ***TOMST*** | ***Air (15 cm)*** | ***1 vs 12*** | ***No mean values*** | ***No mean values*** | ***No mean values*** | ***10 years*** | ***Maryland, USA*** | ***Forest*** | ***BiodiversiTREE*** |
| ***Park et al. 2025*** | ***yes*** | ***yes*** | ***yes*** | ***SensorPush*** | ***Air (84cm)*** | ***1 vs 10*** | ***No mean values*** | ***No mean values*** | ***No mean values*** | ***8 years*** | ***MN, USA*** | ***Forest*** | ***FAB2*** |
| ***Aguirre et al. 2021*** | ***yes*** | ***yes*** | ***yes*** | ***iButton*** | ***Air (12 cm)*** | ***0 vs 8*** | ***0*** | ***+4%*** | ***0*** | ***2 years*** | ***CA, USA*** | ***Grassland*** | ***Bio3D*** |
| ***Whittington et al. 2013*** | ***yes*** | ***no*** | ***no*** | ***iButton*** | ***Soil*** | ***1 vs 32*** | ***0 to -1ºC (depending on season)*** | ***-*** | ***-*** | ***15 years*** | ***MN, USA*** | ***Grassland*** | ***BigBio*** |
| ***Wright et al. 2015*** | ***yes*** | ***yes*** | ***yes*** | ***iButton*** | ***Air (20 cm)*** | ***1 vs 16*** | ***No mean values*** | ***No mean values*** | ***No mean values*** | ***14 years*** | ***MN, USA*** | ***Grassland*** | ***BioCON*** |
| ***Wragg et al. 2015*** | ***yes*** | ***yes*** | ***yes*** | ***iButton*** | ***Air (10 cm) & Soil (1cm)*** | ***1 vs 16*** | ***-0.5ºC in air to -2ºC soil*** | ***No mean values*** | ***-0.15 kPa*** | ***19 years*** | ***MN, USA*** | ***Grassland*** | ***BigBio*** |
| ***Cappelli et al. 2020*** | ***yes*** | ***yes*** | ***no*** | ***iButton*** | ***Air*** | ***1 vs 20*** | ***No mean values*** | ***No mean values*** | ***No mean values*** | ***3 years*** | ***Switzerland*** | ***Grassland*** | ***SwissBEF*** |
| ***Guimarães-Steinicke et al. 2021*** | ***yes*** | ***no*** | ***no*** | ***TLS*** | ***Leaf*** | ***1 vs 8*** | ***0*** | ***-*** | ***-*** | ***4 years*** | ***Germany*** | ***Grassland*** | ***Jena TBE*** |
| ***Wolf et al. 2017*** | ***yes*** | ***no*** | ***no*** | ***Infrared gun*** | ***Soil*** | ***2 vs 16*** | ***-4ºC*** | ***-*** | ***-*** | ***4 years*** | ***CA, USA*** | ***Grassland*** | ***Zavaleta*** |
| ***Huang et al. 2024*** | ***yes*** | ***no*** | ***no*** | ***PT100 Resistor*** | ***Soil (5-15cm)*** | ***1 vs. 60*** | ***+1.5 ºC at night to -4.5 ºC in the summer at mid-day*** | ***-*** | ***-*** | ***18 years*** | ***Germany*** | ***Grassland*** | ***Jena Main*** |
| ***Cowles et al. 2016*** | ***yes*** | ***yes*** | ***yes*** | ***iButton*** | ***Air (10-25 cm)*** | ***1 vs 16*** | ***-1.5 ºC*** | ***-*** | ***-1.8 kPa*** | ***20 years*** | ***MN, USA*** | ***Grassland*** | ***BigBio*** |
| ***Wright et al. 2014*** | ***yes*** | ***yes*** | ***yes*** | ***iButton*** | ***Air (20 cm)*** | ***1 vs 16*** | ***-2ºC*** | ***+13%*** | ***-0.7 kPa*** | ***14 years*** | ***MN, USA*** | ***Grassland*** | ***BioCON*** |
| ***Lundholm et al. 2010*** | ***yes*** | ***no*** | ***no*** | ***Soil temp probe*** | ***Soil (1 cm)*** | ***1 vs 15*** | ***-2.5ºC*** | ***-*** | ***-*** | ***2 years*** | ***Halifax, CA*** | ***Herbaceous Greenroof*** | ***Lundholm*** |
| ***Ramirez et al. 2025*** | ***yes*** | ***yes*** | ***yes*** | ***iButton*** | ***Air (5-10 cm)*** | ***1 vs 3*** | ***0*** | ***0*** | ***0*** | ***4 months*** | ***CA, USA*** | ***Crop System*** | ***Ramirez*** |

***Table S1.*** *We conducted a formal search of the literature for all papers that examined the effect of diversity on humidity or VPD in Web of Science Core Collection and Google Scholar* ("plant diversity" OR "herbaceous diversity" OR "tree diversity" OR "shrub diversity" (All Fields)) AND (microclimate AND (temperature OR “relative humidity” OR VPD) (All Fields)) AND ("diversity gradient" OR "planted diversity" OR "diversity experiment*" OR "complementarity effect" OR "diversity manipulation" OR "biodiversity experiment*" (All Fields)*. After manually reviewing these papers, this yielded 23 published papers (only the 14 with data reported in the main text). All papers and full details are listed here.*

**Supplement References not included in main text:**

*:*

**Cappelli SL. 2020.** Causes and consequences of fungal pathogen infection in grasslands. *Doctoral Dissertation.* **Universität Bern, Bern, Switzerland.**

**Coyne S, Osorio E, Bennett SK,  Kirchgraber A, Parker AD, Nowakowski AJ. 2025.** Bird occurrence and trophic interactions vary across gradients of tree diversity and microclimate in a planted forest. *bioRxiv preprint.* **doi: https://doi.org/10.1101/2025.02.17.637724**

**Wang, M., Blondeel, H., Gillerot, L., Verbeeck, H., Van Coillie, F., Meunier, F., ... & Calders, K. (2025).** Influence of forest canopy structure on temperature buffering in young planted forests with varied tree species compositions revealed by terrestrial laser scanning. *Agricultural and Forest Meteorology,* **371**: 110640.

**Wu D, Pietsch KA, Staab M, Yu M**. **2021**. Wood species identity alters dominant factors driving fine wood decomposition along a tree diversity gradient in subtropical plantation forests. *Biotropica* **53**: 643–657.
